# Circulating MicroRNAs as Predictors of Beta Cell Function in Youth-onset Type 2 Diabetes: The TODAY Study

**DOI:** 10.1210/clinem/dgae376

**Published:** 2024-05-30

**Authors:** Dakota Redling, Shannon Bialak, Laure El ghormli, Steven D Chernausek, Kenneth Jones, Jeanie B Tryggestad

**Affiliations:** University of Oklahoma Health Sciences Center, Oklahoma City, OK 73104, USA; University of Oklahoma Health Sciences Center, Oklahoma City, OK 73104, USA; The Biostatistics Center, George Washington University, Rockville, MD 20852, USA; University of Oklahoma Health Sciences Center, Oklahoma City, OK 73104, USA; University of Oklahoma Health Sciences Center, Oklahoma City, OK 73104, USA; University of Oklahoma Health Sciences Center, Oklahoma City, OK 73104, USA

**Keywords:** youth-onset type 2 diabetes, beta cell function, miRNA

## Abstract

**Aims:**

In the Treatment Options for Type 2 Diabetes in Adolescents and Youth (TODAY) study, an intervention trial followed by an observational phase, half the participants reached the primary outcome [hemoglobin A1c (HbA1c) ≥ 8% for at least 6 months] within 4 years, which was associated with a decrease in C-peptide oral disposition index (oDI). We aimed to identify circulating microRNA (miRNA) species associated with a decline in beta cell function.

**Methods:**

Following a preliminary survey of select participants using nCounter Human v3 miRNA Panel (NanoString Technologies), polymerase chain reaction analyses were carried out for 17 miRNAs from 365 participants from samples at baseline, 24, 60, 96, and 120 months.

**Results:**

Using a backward selection approach, 4 baseline miRNA log2 fold-changes independently predicted treatment failure; however, baseline HbA1c was higher in those with treatment failure. Three baseline miRNA log2 fold-changes remained significant predictors of this C-peptide oDI decline ≥20% (*P* < .05). Increased levels of miRNA-155 [odds ratio (OR): 1.2, 95% confidence interval (CI): 1.1-1.4] and miRNA-130b (OR:1.3, 95% CI: 1.0-1.7) were associated with oDI decline, while decreased levels of miRNA-126 (OR: 0.6, 95% CI: .4-.8) were associated with oDI decline. miRNA-122 was negatively correlated with C-peptide oDI at baseline and 24 months (R = 0.22, *P* < .01 and R = 0.19, *P* < .01, respectively) and positively correlated with proinsulin at baseline, 24, and 60 months (R = 0.26, *P* < 0.01, R = 0.26, *P* < .01, R = 0.18, *P* < .01, respectively).

**Conclusion:**

The miRNA species associated with beta cell function are associated with alterations in cellular metabolism and apoptosis, suggesting that differences in baseline abundance may serve as circulating markers of beta cell dysfunction and provide potential mechanistic insights into the aggressive nature of youth-onset type 2 diabetes.

The incidence of type 2 diabetes in youth has risen dramatically in the past 20 years. The SEARCH for Diabetes in Youth Study showed that the prevalence of type 2 diabetes in youth (10-19 years) more than doubled between 2002 and 2018 with an annual increase in cases of 5.3% per year. In fact, the incidence of type 2 diabetes in youth between the ages of 15 and 19 years now exceeds that of type 1 diabetes (1). It is estimated that the prevalence of type 2 diabetes in youth will increase by 700% by the year 2060 (2).

Type 2 diabetes is an especially aggressive disease when it presents in youth. From the Treatment Options for Type 2 Diabetes in Adolescents and Youth (TODAY) study, an intervention trial followed by an observational phase, almost half the participants with youth-onset type 2 diabetes required intensification of treatment due to inability to maintain treatment goals or metabolic decompensation (3). Moreover, diabetes-related complications were seen in 60% of the participants by 10 years on average—a sobering testimony to the seriousness of type 2 diabetes in youth (4). The aggressive nature of the disease is due to rapid, pronounced beta cell failure with complications resulting from hyperglycemia and insulin resistance (5).

The ability to timely predict which youth will develop treatment failure or have a decline in beta cell function has been challenging. In the TODAY study, baseline higher hemoglobin A1c (HbA1c) and lower C-peptide oral disposition index (oDI), along with maternal history of diabetes, predicted which youth would progress to treatment failure, defined as HbA1c ≥ 8% over a period of 6 months or inability to wean from insulin after metabolic decompensation (6); however, by the time the glycemic predictors are evident, the disease has already progressed significantly. Additionally, these measures provide no insight into the mechanisms that lead to beta cell dysfunction. To better understand the mechanistic underpinnings, genetic risk scores have been developed, but unfortunately, their ability to predict youth-onset type 2 diabetes is less than simple anthropometrics and family history (7). Thus, there is a need to identify biomarkers that not only predict the progression of youth-onset type 2 diabetes but also predict the development of youth-onset diabetes.

MicroRNA (miRNA) are small, noncoding RNAs that bind to the 3′ untranslated region of protein-coding mRNAs, modulating translation by degrading or repressing translation of target mRNAs. Recent reports demonstrate that changes in miRNA abundance are predictors of progression to adult-onset type 2 diabetes and provide a mechanistic link to the development of diabetes-related complications (8-13). Because miRNAs are released into circulation, assessment of circulating miRNAs offers the opportunity to better understand the pathogenesis of type 2 diabetes and its complications and to identify biomarkers that predict or identify disease progression (14).

While miRNAs predicting the development of type 2 diabetes have been explored in adults, no study has examined the role of miRNAs in relation to beta cell failure in youth-onset type 2 diabetes. Biomarkers of disease progression and microvascular complications, such as adhesion molecules and inflammatory cytokines, have been assessed, but to date, none has been proven to reliably predict beta cell function. The TODAY study offers a unique opportunity to address the roles of circulating miRNAs in youth-onset type 2 diabetes as the participants have been deeply phenotyped regarding metabolic, physical, and psychosocial parameters, with multiple measures obtained for more than a decade. Thus, the objective of this study was to identify miRNA species that would predict beta cell function during disease progression.

## Methods

The TODAY study design has been described previously (3). Briefly, 699 participants, 10 to 17 years old, diagnosed with type 2 diabetes by American Diabetes Association criteria, with duration 2 years or less, body mass index (BMI) ≥ 85th percentile for age and sex, were enrolled across 15 clinical centers in the United States (15). Participants were randomized to either metformin alone, metformin with rosiglitazone, or metformin plus a lifestyle intervention program and followed longitudinally for 2 to 6 years. After the clinical trial (2004-2011), an observational follow-up study (TODAY2) was conducted in 2 phases (phase 1, 2011-2014; phase 2, 2014-2020). During the first phase, participants received standard diabetes care. In the second phase, clinical care was no longer provided through the study, but annual visits continued for the collection of biologic specimens, assessment of microvascular and macrovascular complications, and capture of demographic and health data. All participants provided consent or assent as appropriate. All phases of the study were approved by the local Institutional Review Boards of the 15 clinical centers. Fasting measurements of HbA1c, insulin (RRID:AB_2915954), and proinsulin (RRID:AB_2891152), as well as measurements of C-peptide (RRID:AB_3083552) and glucose from oral glucose tolerance tests were obtained and analyzed at the TODAY Central Biochemistry Laboratory (Northwest Lipid Metabolism and Diabetes Research Laboratories, University of Washington, Seattle, WA) according to standardized procedures (3, 5). Treatment failure was defined as a persistently elevated HbA1c (≥8%) over a period of 6 months or inability to wean from insulin after metabolic decompensation. C-peptide oDI, a measure of beta cell function, was defined as the product of insulin sensitivity (1/fasting insulin) multiplied by the C-peptide index (ratio of the incremental C-peptide and glucose responses over the first 30 minutes of the oral glucose tolerance test) (5). For further classification of beta cell function, the cohort was divided into those with a 20% or greater decrease in the C-peptide oDI in the first 6 months of the clinical trial and those who did not have a 20% decrease in C-peptide oDI. The cut-point of 20% was based on the finding that those who experienced treatment failure had about a 20% reduction in C-peptide oDI within 6 months (5).

A broad-based screen was used initially to identify miRNAs that were potentially differentially expressed in youth who experienced treatment failure in the clinical trial phase of the TODAY study (HbA1c ≥ 8% for at least 6 months or inability to wean from insulin after a metabolic decompensation, n = 6) compared to those who maintained glycemic control (n = 10). Participants were matched for diabetes duration, race/ethnicity, and sex. Only participants randomized to the metformin-only arm were used for screening, and they had to have a sample at both their baseline visit and the end of study visit (after an average of 8 ± 2 years of follow-up).

Total RNA was extracted from 400 µL of plasma using the Qiagen miRNeasy Serum/Plasma Advanced Kit according to the manufacturer's directions. The RNA was then concentrated using the Zymo RNA Clean & Concentrator kits according to the recommendations for the concentration of small RNAs. The miRNA profiling was done using the nCounter Human v3 miRNA Panel (NanoString Technologies). The nCounter Human v3 miRNA Panel (NanoString Technologies) contains 827 well-characterized miRNAs. A total of 3 µL of concentrated RNA was used in the recommended protocol.

### Quantitative PCR Analysis

Based on the results of the Nanostring miRNA profiling and literature review, 17 miRNA species (9 from the Nanostring profile and 8 from literature review) were selected for subsequent quantitative PCR (qPCR) analysis in plasma [Table S1 (16)]. The selected miRNAs were analyzed in 365 of the 699 individuals within the TODAY study for which a baseline plasma sample was available. Additional plasma samples at visits 24, 48, 60, 96, and 120 months in the trial were used to assess changes in circulating miRNA over time. RNA was extracted from 200 µL of plasma from each participant at each time point. cDNA was made from the RNA using the Taqman Advanced miRNA cDNA synthesis kit (Thermo Fisher Scientific, Waltham, MA) per the included instructions. qPCR was carried out in a 384-well format for each sample in triplicate using Taqman Advanced miRNA Assays (Thermo Fisher Scientific). Comparison of the 365 participants included in the analysis sample to the other 334 TODAY study participants (out of the 699 randomized) shows that those included in this sample had a slightly shorter duration of type 2 diabetes diagnosis at baseline (mean ± SD 7.4 ± 5.7 vs 8.3 ± 6.0 months, *P* = .03) but were otherwise not different in regard to sex, race/ethnicity, baseline BMI and HbA1c, or baseline age.

## Data Analysis

The NanoString results were processed with a custom pipeline using Python and R. The target counts for each sample were scaled by 1 million for comparison. Each miRNA within the sample was normalized by the sample's total miRNA expression. Samples were pooled into control or treatment groups and normalized. Fold change for samples was then calculated as log2 from the group mean. The data was passed using R for a fit linear model between control and treatment then ANOVA for variance. The *P*-value was adjusted using Benjamini and Hochberg correction. Principal component analysis plots were generated for all samples. Targets were selected for further analyses where the *P*-value was less than .05.

For the qPCR validation, the samples were normalized to the geometric mean of all samples. 2^−ΔΔCt^ was used to calculate the fold-change in the individuals with treatment failure as compared to those who did not experience treatment failure. Cyclic threshold values below the detectable range were imputed as 40 for the calculation of the geometric mean but were not included in fold-change analysis as outliers, which were defined as a fold-change greater than 100.

Descriptive statistics reported include frequencies, percentages, means, and SDs. Quantitative characteristics at baseline were compared between sex and race/ethnicity subgroups using the Wilcoxon rank sum test and Kruskal–Wallis test. The associations between circulating miRNAs levels at baseline and the treatment failure and beta cell function outcomes were assessed with logistic regression models, and the trapezoidal rule method was used to calculate area under the curves (AUCs). Separate multivariable logistic regression models that included all 17 miRNAs species were developed with the use of a backward elimination procedure. The final multivariable models retained the selected covariates significant at *P*-values <.05 along with a priori determined demographic and metabolic characteristics (age at baseline, race/ethnicity, sex, duration of diabetes at baseline, and baseline BMI). The odds ratios (ORs) and 95% confidence intervals (CIs) are presented. Similar univariable and multivariable regression models were used to predict the ≥20% reduction in C-peptide oDI in the first 6 months outcome instead of treatment failure. Due to data skewness, miRNAs fold-changes are given in a base log2-transformation and analyzed on that log2 scale for testing. Linear mixed models were used to evaluate mean differences in miRNA over time according to treatment failure group. Spearman correlations were used to measure the degree of association between the fold-change of circulating miRNAs and beta cell or glycemic measures at a given time point. *P*-values <.05 were considered significant, and no adjustment for multiple testing was performed as these analyses are considered exploratory. Analyses were performed using SAS 9.4 (SAS Institute, Cary, NC).

## Results

### Nanostring Profiling and Selection of miRNAs for the Study

**Figure 1.**
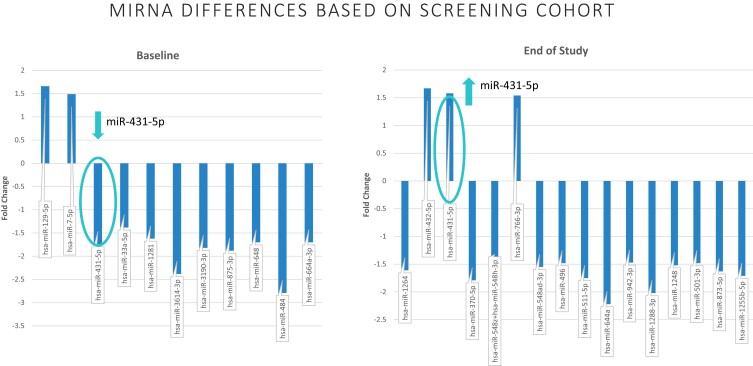
Nanostring miRNA analysis. From the initial screening including 11 participants with no treatment failure and 6 with treatment failure by the end of the clinical trial, 11 miRNA species were different at baseline and 16 were different at the end of study visit (approximately 8 years later) (*P* < .05 for all). Only miRNA-431-5p was significantly different at both time points but with different direction in abundance. Abbreviations: miRNA, microRNA.

From the miRNA profiling, 11 miRNAs were differentially expressed in the participants with treatment failure compared to those who maintained glycemic control at baseline (*P* < .05). At the end of study, approximately 8 years later, 16 miRNA species were differentially expressed (*P* < .05). Only miRNA-431-5p differed in abundance at baseline and end of study, with lower abundance at baseline in the group with treatment failure but higher abundance in that group at the end of the study. (Fig. 1) From these data as well as a review of the literature (8, 10, 11, 13, 17-19), 17miRNA species were selected for analysis in the larger group.

### Prediction of Treatment Failure by miRNA Abundance

In the larger cohort, 168 of 365 individuals (46%) failed treatment (HbA1c ≥ 8% over 6 months or inability to wean from insulin after a decompensation). Diabetes duration was longer and HbA1c at baseline was higher in the group with treatment failure. (Table 1) There was no difference in race/ethnicity, sex, or BMI between the groups.

**Table 1.** Demographic information

	Overall (n = 365)	Glycemic failure (n = 168, 46%)	No glycemic failure (n = 197, 54%)	Decrease in C-peptide oDI ≥20% in the first 6 months (n = 150, 44%)	No ≥20% decrease in C-peptide oDI in the first 6 months (n = 195, 56%)
Age (years)	14 ± 2.0	**14.1 ± 2.0**	13.9 ± 1.9	14.1 ± 1.9	13.9 ± 2.0
Female (%)	61.9	58.3	65.0	60.0	64.6
Type 2 diabetes duration (months)	7.4 ± 5.7	**8.2 ± 6.2**	6.8 ± 5.2	7.3 ± 5.6	7.3 ± 5.6
Race-ethnicity (%)					
Non-Hispanic Black	35.6	39.3	32.5	34.7	34.9
Hispanic	43.8	45.8	42.1	46.7	43.1
Non-Hispanic White	20.5	14.9	25.4	18.7	22.0
Treatment group (%)					
Metformin alone	33.1	33.3	33.0	33.3	33.3
Metformin + rosiglitazone	32.9	28.6	36.5	28.0	37.4
Metformin + intensive lifestyle	34.0	38.1	30.5	38.7	29.2
Anthropometrics					
Height (cm)	96.3 ± 26.3	165.6 ± 9.8	164.9 ± 9.5	164.7 ± 10.0	165.4 ± 9.4
Weight (kg)	165.2 ± 9.6	96.7 ± 26.5	95.9 ± 26.2	95.2 ± 25.0	95.8 ± 27.0
BMI (kg/m^2^)	35.0 ± 8.2	34.9 ± 7.7	35.0 ± 8.5	34.8 ± 7.6	34.8 ± 8.6
Glycemic metabolism					
HbA1c (%)	6.1 ± 0.8	**6.4 ± 0.8**	5.8 ± 0.6	6.0 ± 0.7	6.1 ± 0.8
Glycemic failure (%)	—	**—**	—	**52.0**	38.8
C-peptide oDI (mL/µU × ng/mL per mg/dL)		**0.001** **[0.001-0.003]**	0.003 [0.002-0.005]	0.003 [0.001-0.005]	0.002 [0.001-0.004]
Decrease in C-peptide oDI ≥20% in the first 6 months (%)	—	**54.2**	40.9	—	—

Data are mean ± SD, median [interquartile range], or %. Bold indicates *P*-value < .05 for the comparison between groups. C-peptide oDI was not available for participants who did not complete an oral glucose tolerance test (20 of the 365 participants).

Abbreviations: BMI, body mass index; HbA1c, hemoglobin A1c; oDI, oral disposition index.

The miRNA abundances at baseline are reported in Table S2 (16). Some abundances varied with the sex and race/ethnicity of the participants. Baseline abundance of miRNA-122-5p was lower in non-Hispanic Black participants when compared to the Hispanic participants (*P* = .001) but not different from non-Hispanic White participants. Baseline abundance of miRNA-194 was higher in Hispanic participants when compared to the non-Hispanic Black and non-Hispanic White participants (*P* = .011, and *P* = .027, respectively). Baseline abundance of miRNA-431-5p was lower in non-Hispanic Black participants when compared to the non-Hispanic White participants (*P* = .021) but not different from Hispanic participants. miRNA-15b was higher in abundance at baseline in the females compared to the males (*P* = .030).

**Figure 2.**
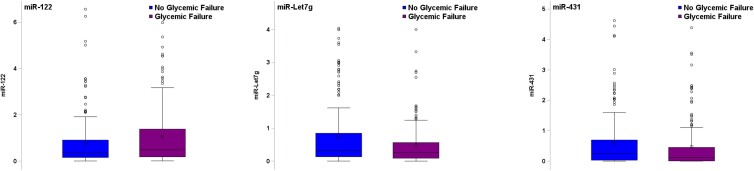
miRNAs that independently predict treatment failure. Treatment failure was defined as a persistently elevated HbA1c (≥8%) over a period of 6 months or inability to wean from insulin after metabolic decompensation. At baseline, miRNA-122, let7g, and 431 all predicted treatment failure (*P* = .04, .04, and .01, respectively). Abbreviations: HbA1c, hemoglobin A1c; miRNA, microRNA.

**Figure 3.**
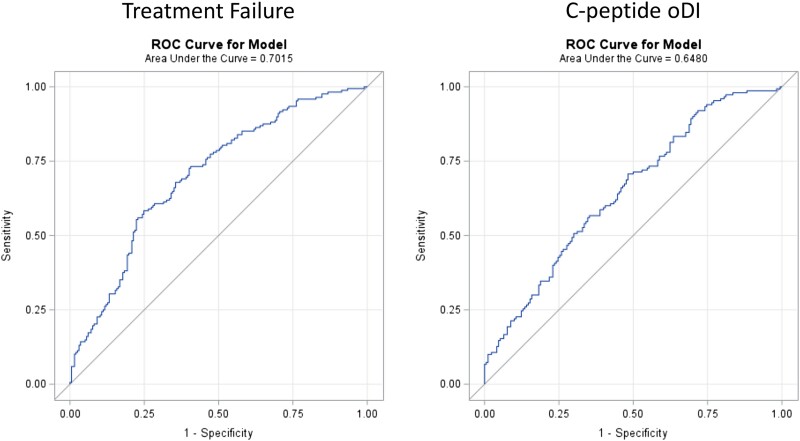
Receiver operating characteristic curve for multivariable models for predicting glycemic failure and C-peptide oDI decline. The models including the combination of miRNAs and demographic variables improved the prediction for both treatment failure and decline in C-peptide oDI as compared to the single miRNA species. Abbreviations: miRNA, microRNA; oDI, oral disposition index.

Examining differences in miRNA abundance at baseline, a higher circulating abundance of miRNA-122-5p predicted treatment failure (*P* = .04) while a lower circulating abundance of miRNA-431-5p and miRNA-let-7g-5p predicted treatment failure (*P* = .01 and *P* = .04, respectively), in separate unadjusted logistic regression models [Fig. 2, Table S3 (16)]. The AUC range for these miRNAs was between 0.56 and 0.57, indicating weak ability to predict failure when evaluated individually. In a multivariable logistic regression model using a backward selection approach to identify a combination of miRNAs at baseline that would predict treatment failure, miRNA-194-5p, miRNA-15b-5p, miRNA-let 7g-5p, and miRNA-431-5p independently predicted treatment failure after controlling for age, sex, race/ethnicity, BMI, and diabetes duration (Table 2). Indeed, 1-unit increases in log2 miRNA-194-5p and log2 miRNA-15b-5p fold-changes at baseline corresponded to 50% and 20% higher odds of glycemic failure (OR: 1.5, 95% CI: 1.2-1.9 and OR: 1.2, 95% CI: 1.1-1.3, respectively). In addition, 1-unit decreases in log2 miRNA-let 7g-5p and log2 miRNA-431-5p fold-changes at baseline corresponded to higher odds of glycemic failure by 30% and 10% (OR: 0.7, 95% CI: .5-.8 and OR: 0.9, 95% CI: .8-1.0, respectively). The AUC for the multivariable model using the combination of miRNAs was improved to 0.70, suggesting better model discrimination capability compared to the univariable models with the single miRNAs (Fig. 3).

**Table 2.** Combination of L2FC at baseline associated with treatment failure

	OR	Lower 95% CI	Upper 95% CI	Wald chi-square
L2FC194	1.52	1.21	1.89	13.3
L2FC15b	1.19	1.09	1.29	15.1
L2FC7g	0.68	0.54	0.85	11.7
L2FC431	0.86	0.76	0.98	5.5
Age (years)	1.12	0.99	1.26	3.1
T2D duration (months)	1.05	1.01	1.09	5.9
Female (ref: male)	0.80	0.50	1.28	0.9
Hispanic (ref: NHW)	1.88	1.01	3.49	0.8
NHB (ref: NHW)	2.30	1.19	4.48	4.1
BMI	0.98	0.95	1.01	2.2

Multivariable logistic regression model predicting the odds of treatment failure (glycemic failure). The ORs with corresponding 95% CIs are provided for each covariate. The Wald chi-square corresponds to the magnitude of the association, testing the null hypothesis that an individual predictor's regression coefficient is 0 given the other predictor variables are in the model.

Abbreviations: BMI, body mass index; CI, confidence interval; L2FC, log2 fold-changes; NHB, Non-Hispanic Black; NHW, Non-Hispanic White; OR, odds ratio; TD2, type 2 diabetes.

**Figure 4.**
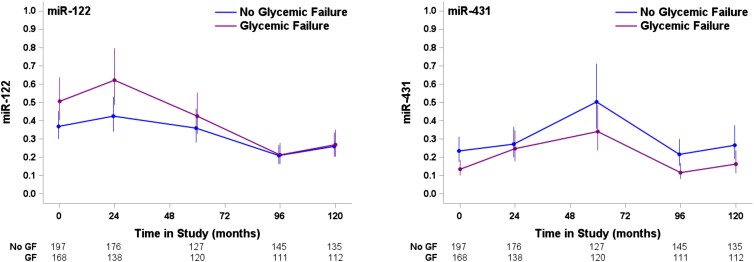
miRNA abundance over time predicts treatment failure. Circulating miRNA was assessed at baseline, 24 months, 60 months, 96 months, and 120 months. Over time, miRNA-431 abundance continued to predict treatment failure (*P* = .01); however, abundance of miRNA-122 over time was not significant after adjustment for baseline characteristics (*P* = .07). Abbreviations: miRNA, microRNA.

Differences in miRNA abundance over time between those who experienced treatment failure and those who did not were also assessed. Overall, mean differences in miRNA-122-5p observed at baseline between the 2 groups were attenuated when evaluated in linear mixed model adjusted for baseline age, sex, race/ethnicity, duration of diabetes, and baseline BMI (*P* = .07) whereas the differences in miRNA-431-5p observed at baseline were maintained over time (*P* = .01) (Fig. 4). No difference by treatment failure over time in miRNA-let-7g-5p, miRNA-194-5p, or miRNA-15b-5p was noted.

### Prediction of Beta Cell Function by miRNA Abundance

The ability of the miRNA abundance to predict specific indices of beta cell function was also examined. In the cohort, 150 participants (43%) had a reduction in C-peptide oDI in the first 6 months of ≥20% (Table 1). Higher miRNA-122-5p and miRNA-155-5p abundance at baseline predicted a ≥ 20% decrease in C-peptide oDI in the first 6 months (*P* = .01 for both) in a univariable logistic regression model with a resulting AUC of 0.58 and 0.56, respectively [Table S4 (16)]. No other miRNA baseline abundance was related to the beta cell function outcome. In a multivariable logistic regression model using a backward selection, miRNA-122-5p was no longer significant, but instead, miRNA-155-5p remained significant; in combination with miRNA-126-3p and miRNA-130b-3p abundance at baseline, all three predicted the decrease in C-peptide oDI after controlling for age, sex, race/ethnicity, BMI, and diabetes duration (Table 3). For every 1-unit increase in log2 miRNA-155-5p and log2 miRNA-130b-3p fold-changes at baseline, there was a corresponding 20% and 30% increase in the odds of C-peptide oDI reduction at 6 months (OR: 1.2, 95% CI: 1.1-1.4 and OR: 1.3, 95% CI: 1.0-1.7, respectively). In addition, for every 1-unit decrease in log2 miRNA-126-3p fold-change at baseline, there was a corresponding 40% increase in the odds of C-peptide oDI reduction at 6 months (OR: 0.6, 95% CI: .4-.8). The AUC for the multivariable model was 0.65 (Fig. 3), which was improved from the single-variable models.

**Table 3.** Combination of L2FC at baseline associated with 20%+ decline in C-peptide oDI during the first 6 months of the study

	OR	Lower 95% CI	Upper 95% CI	Wald chi-square
L2FC122	1.11	0.98	1.26	2.9
L2FC126	0.60	0.44	0.81	11.1
L2FC155	1.21	1.07	1.38	8.7
L2FC130b	1.32	1.02	1.70	4.6
Age (year)	1.08	0.95	1.22	1.3
T2D duration (month)	1.01	0.97	1.05	0.09
Female (ref: male)	0.99	0.60	1.61	0.004
Hispanic (ref: NHW)	1.24	0.67	2.29	0.02
NHB (ref: NHW)	1.43	0.74	2.78	0.9
BMI	0.99	0.96	1.02	0.25

Multivariable logistic regression model predicting the odds of a 20%+ C-peptide oDI decline in the first 6 months. The ORs with corresponding 95% CIs are provided for each covariate. The Wald chi-square corresponds to the magnitude of the association, testing the null hypothesis that an individual predictor's regression coefficient is 0 given the other predictor variables are in the model.

Abbreviations: BMI, body mass index; CI, confidence interval; L2FC, log2 fold-changes; NHB, Non-Hispanic Black; NHW, Non-Hispanic White; oDI, oral disposition index; OR, odds ratio; TD2, type 2 diabetes.

In addition to C-peptide oDI, associations of other measures of beta cell function (proinsulin, proinsulin/insulin ratio) with circulating miRNAs were also examined using correlation analysis at select time points. miRNA-122-5p abundance was negatively correlated with C-peptide oDI at baseline and at the 24-month time point (R = −0.22, *P* < .001 and R = −0.19, *P* = .001, respectively) and was positively correlated with circulating proinsulin levels at baseline, 24, and 60 months (R = 0.26, *P* < .001, R = 0.26, *P* < .001, and R = 0.18, *P* = .004, respectively) [Figure S1 (16)]. Weaker positive correlations were noted between the abundance of miRNA-122-5p and the proinsulin/insulin ratio at each time point (all Rs < 0.12). No other significant correlations were observed between the measures of beta cell function and additional circulating miRNAs (data not shown).

## Discussion

In this study of youth with type 2 diabetes, the combined circulating abundances of miRNA-194-5p, miRNA-15b-5p, miRNA-let 7g-5p, and miRNA-431-5p at baseline predicted which individuals would require intensification of treatment to maintain treatment goals at a level similar to that of a baseline HbA1c ≥ 6.2% (20). In addition, the combined expression levels of miRNA-126-3p, miRNA-155-5p, and miRNA-130b-3p specifically predicted a decline in beta cell function. Wander and colleagues examined a smaller cohort of the TODAY study finding that a 12% lower abundance of miR-4306 at baseline predicted treatment failure at 1 year (21) but did not examine the relationship with beta cell function. In the present analysis, the time to treatment failure was throughout the study, thus increasing the power to detect baseline miRNAs that would predict future treatment failure. To our knowledge, this is the first study to identify groups of miRNAs that together at baseline predict beta cell function.

miRNA-126-3p in the TODAY cohort was negatively associated with beta cell function using the multivariable model, consistent with lower abundance in adults who develop type 2 diabetes (19). Also, higher miRNA-194-5p in the multivariable model predicted treatment failure in the TODAY cohort, and in adult cohorts, miRNA-194-5p has been shown to predict the incidence of type 2 diabetes (17). In vitro, a reduction in miRNA-194-5p results in increased phospho-AKT and thus increased insulin signaling (22). Therefore, the increase in miRNA-194-5p may reduce phospho-AKT and thus insulin action in the target cells. Other miRNAs, such as miR-15a, −29b, −150, and −375, which predicted the development of diabetes in adults (18, 19), were not found to be different in our screening analysis and thus were not examined further.

From a meta-analysis of adults with type 2 diabetes, circulating abundance of miRNA-122-5p was positively correlated with fasting blood glucose and insulin resistance (23). In TODAY participants, miRNA-122-5p was positively associated with proinsulin levels and negatively associated with C-peptide oDI, both of which reflect beta cell dysfunction. These observations are potentially explained by anticipated effects of miRNA-122-5p. miRNA-122-5p targets pyruvate kinase M, potentially suppressing insulin secretion (24). In addition, miRNA-122-5p increases oxidative stress and apoptosis in a beta cell line by acting on the Akt/PI3K pathways (25).

The combination of abundances of the specific miRNAs in the TODAY study proved to be superior at predicting treatment failure over the individual miRNA species based on the AUC. The combination also demonstrated a comparable prediction of treatment failure to glycemic variables that predicted treatment failure in the TODAY study (6). In the TODAY cohort, the variation in fasting plasma glucose during the first year of the study had an AUC of 0.7 with the baseline HbA1c having an AUC of 0.66 (6), and the combination of miRNAs had an AUC of 0.7 as well. In the CARDIOPREV study, the combination of miRNAs proved to be superior at predicting progression to type 2 diabetes in adults; thus using the combination of miRNAs in addition to the glycemic variability may increase the ability to predict treatment failure in youth-onset type 2 diabetes.

Interestingly, there was little overlap between the miRNAs that predicted treatment failure and those that predicted a decline in beta cell function. While beta cell failure is clearly linked to treatment failure, insulin resistance through pathways such as inflammation and oxidative stress also contribute toward treatment failure. The miRNAs identified as predicting treatment failure have been implicated in these pathways (26-29).

The association of miRNA-431-5p abundance with subsequent treatment failure is novel and is potentially unique to youth-onset type 2 diabetes. Lower levels of miRNA-431-5p were first seen in the screening profile and subsequently validated by PCR. While no studies have specifically examined miRNA-431-5p in relation to beta cell function or treatment failure, it does target insulin receptor substrate 2 (30). This early decrease in miRNA-431-5p may be a protective mechanism to increase insulin receptor substrate 2 abundance in the setting of insulin resistance in youth-onset type 2 diabetes. While lower baseline abundance was associated with treatment failure, in the screening of participants at the end of the study, the abundance of miRNA-431-5p was higher in those with treatment failure. This may suggest that, over time, an increase in abundance may predict the progression to diabetes-related complications as increasing abundance of miRNA-431-5p was associated with worse disease and promoted endothelial cell proliferation in vitro (31).

The current study has several strengths. This is the largest study of circulating miRNAs to date from an extensively phenotyped group of individuals with miRNA data over a 10-year period. The miRNAs chosen for validation were based on the unbiased broad screening approach using NanoString technology. A few limitations are acknowledged as well. The exact mechanisms of the miRNAs are not identified in the present study, but it does identify potential pathways and mechanisms to explore in youth-onset type 2 diabetes. The number of miRNA species validated was limited, but efforts were made to select those that were highly abundant with the highest fold-change from the screening analysis.

In conclusion, we have identified miRNAs that predict treatment failure and beta cell function decline in youth-onset type 2 diabetes as well as those that are associated with markers of beta cell function. Circulating miRNAs may prove to be predictors of beta cell failure but may also provide key insights into potential mechanisms underlying beta cell failure. Future work should examine the potential mechanisms that are implicated by these miRNA species as well as the ability of newer therapies to alert their expression.

## Data Availability

Data collected for the TODAY study are available to the public through the NIDDK Repository (TODAY https://repository.niddk.nih.gov/studies/today, TODAY2 https://repository.niddk.nih.gov/studies/today2/).

## Abbreviations

AUC: area under the curve
BMI: body mass index
CI: confidence interval
HbA1c: hemoglobin A1c
miRNA: microRNA
oDI: oral disposition index
OR: odds ratio
qPCR: quantitative PCR
TODAY: Treatment Options for Type 2 Diabetes in Adolescents and Youth
